# Editorial: Highlights in educational psychology: teacher-student relationship

**DOI:** 10.3389/fpsyg.2024.1529198

**Published:** 2024-12-04

**Authors:** Claudio Longobardi, Elisabetta Sagone, Alberto Crescentini

**Affiliations:** ^1^Department of Psychology, University of Turin, Turin, Italy; ^2^Department of Educational Sciences, University of Catania, Catania, Italy; ^3^Department of Education and Learning, University of Applied Sciences and Arts of Southern Switzerland, Manno, Switzerland

**Keywords:** teacher-student relationship, educational psychology, cultural differences, achievement, instrument

## Introduction

The teacher-student relationship, first significantly studied by Pianta (1992), has been shown to play a critical role in students' psychological and academic success. Drawing on attachment theory, teachers are seen as “*ad hoc*” attachment figures, providing a “secure base” that fosters emotional wellbeing, integration within the classroom, and better learning outcomes (Prino et al., 2023; Verschueren and Koomen, 2012; Farmer et al., 2011). This relationship, characterized by high levels of closeness and low conflict, shapes developmental outcomes across schooling levels and cultures (Longobardi et al., 2019, 2023; Spilt et al., 2011; Fabris et al., 2022, 2023).

This Research Topic deepens the role of teacher-student relationships in educational outcomes and examines how cultural contexts, student characteristics, and even the university setting affect these relationships. It includes 25 studies from various regions, demonstrating the global interest in understanding and enhancing these dynamics.

## Teacher-student relationship in diverse cultural contexts

Research indicates that perceived teacher support positively impacts student adjustment, even across cultures. Tang and Zhu explored the mediating role of teacher support in China, finding that it strengthens the link between academic self-efficacy, grit, and students' psychological wellbeing among English language learners. Similarly, Wang C. et al. reported that perceived math teacher support reduces math anxiety through improved student-teacher relationships and math self-efficacy, though only among male students. These findings underline the need to consider gender differences in how teacher support affects student outcomes.

Ulmanen et al.'s longitudinal study in Finland also underscores the impact of teacher support in reducing burnout and increasing engagement among primary and secondary students. In another Chinese study, Xu et al. found that perceived teacher support indirectly fosters student engagement by fulfilling psychological needs.

These studies collectively highlight that teacher-student relationships and perceived support promote greater school engagement and adjustment across diverse cultural settings.

## Characteristics of students that enhance teacher-student relationships

Student characteristics also influence teacher-student dynamics. Studies suggest that certain student behaviors and qualities affect teacher perceptions and interaction styles. Shi et al. investigated which traits make preschool children “teacher's pets” and found proactive, enthusiastic, and obedient children were favored, while vulnerable or anxious children were less so. Understanding how student traits impact teacher perception and relationship quality could help educators develop strategies to ensure all students benefit equally from positive teacher interactions.

## The teacher-student relationship in the university context

Most research on teacher-student relationships has focused on the primary and secondary school levels. However, recent studies have begun to explore these dynamics in universities. Yin and Lou identified self-regulation as a mediating factor between teacher support and online learning engagement among Chinese university students. Wang G. et al., drawing on attachment theory, found that reciprocity in teacher-student interactions fosters a sense of belonging to the university, with social group attachment further strengthening this effect.

Another study by Pan and Yao demonstrated that teacher support and positive teacher-student relationships significantly influence student engagement in China. Similarly, Li's research showed that promoting positive teacher-student relationships and a growth mindset enhances enjoyment and engagement in language learning among college students.

## Teacher-student relationship's impact on teacher wellbeing

Research is also uncovering the impact of teacher-student relationships on teachers' wellbeing. Zheng et al., for instance, found that positive teacher-student relationships mediate the relationship between gratitude, job crafting, and wellbeing among Chinese English teachers, who often face high burnout risks. In Shanghai, Wang X. et al. linked positive teacher-student relationships to reduced emotional exhaustion, finding that such relationships enhance teachers' motivation, a protective factor against burnout.

## Research instruments

This Research Topic also highlights tools that can aid in researching and enhancing teacher-student relationships. For example, Poulou et al. presented the Classroom Strategies Assessment System's usability among Greek educators. Additionally, Ragni et al. validated the Self-compassion Scale for Italian special needs teachers, which could help practitioners foster positive classroom dynamics.

## Conclusion

The teacher-student relationship remains a vibrant area of research with implications for both student outcomes and teacher wellbeing. This Research Topic offers significant studies. The origin of the articles, mainly Chinese, represents a weak point for this monographic issue also considering the enormous diversity present in Chinese contexts, emphasizing the importance of cross-cultural perspectives to understand how these relationships vary across contexts. Future research should aim for longitudinal studies to explore causality and consider complex models incorporating individual and social context factors. The university context, though relatively underexplored, presents a new frontier for understanding teacher-student dynamics and their unique impacts on learning and adjustment.

We extend our gratitude to all contributing researchers and wish readers insightful engagement with this Research Topic.
